# IV Vitamin C with Chemotherapy for Cisplatin-Ineligible Bladder Cancer Patients: A Forgotten Group

**DOI:** 10.3390/antiox15101318

**Published:** 2026-10-13

**Authors:** John A. Taylor, Rahul A. Parikh, Qi Chen, Benjamin L. Woolbright, Ping Chen, Junqiang Dai, Mihaela Sardiu, Joya He, Jeanne Drisko

**Affiliations:** 1 Department of Urology, University of Kansas Medical Center, Kansas City, KS 66160, USA; 2 Department of Cancer Biology, University of Kansas Medical Center, Kansas City, KS 66160, USA; 3 Department of Internal Medicine, Division of Medical Oncology, University of Kansas Medical Center, Kansas City, KS 66160, USA; 4 Department of Pharmacology, Toxicology and Therapeutics, University of Kansas Medical Center, Kansas City, KS 66160, USA; 5 Department of Biostatistics and Data Science, University of Kansas Medical Center, Kansas City, KS 66160, USA; 6 Dubu Research, Philadelphia, PA 19104, USA; 7 Department of Internal Medicine, Integrative Medicine Research, University of Kansas Medical Center, Kansas City, KS 66160, USA

**Keywords:** vitamin C, muscle invasive bladder cancer, neoadjuvant therapy, combination therapy, pathological downstaging

## Abstract

**Purpose:** Neoadjuvant cisplatin-based chemotherapy (NAC) is considered the standard of care (SOC) for patients with muscle-invasive bladder cancer (MIBC). However, approximately 50% of patients are cisplatin-ineligible (CI) and often proceed directly to surgery. We initiated a phase Ib/IIa trial to evaluate high-dose intravenous ascorbate (IVC) with gemcitabine/carboplatin (Gem/Carbo) as NAC for patients with CI-MIBC (NCT04046094). **Patients and Methods:** Patients with newly diagnosed CI-MIBC received a single cycle of Gem (1000 mg/m^2^ D1, 8)/Carbo (AUC 5 d 1) and IVC (25–125 g titrated to 350–400 mg/dL blood concentrations 2 × per week for 21 days) followed by definitive cystectomy. The primary outcome was pathological downstaging (<ypT2N0Mx) at cystectomy. Patient-reported quality of life (QOL) was assessed using the FACT-BI at enrollment, end of treatment, and 6 weeks after cystectomy. **Results:** Sixteen participants were enrolled, and 12 completed the protocol. The mean age was 76. Pathological downstaging was present in four patients (33%), with 3/4 patients having a pathologic complete response (pCR, ypT0N0Mx). The treatment was well tolerated. At a mean follow-up of 32.3 months (SD 18.9, range 6.6–63.8), four patients without response recurred and died of disease, and three with response had recurred, with one passing from disease. The QOL FACT-Bl did not change from baseline to the end of treatment to 6 weeks post-cystectomy. **Conclusions:** Neoadjuvant, single-cycle Gem/Carbo + IVC had significant activity in patients with CI-MIBC with a response rate (RR) comparable to historical outcomes of SOC therapy in this early-phase trial. Larger trials are warranted to confirm these findings and to identify biomarkers for response.

## 1. Introduction

Bladder cancer (BCa) is the fifth most common cancer in the United States. Nearly one-third of patients with BCa are diagnosed with muscle-invasive bladder cancer (MIBC). Approximately 50 percent of patients with MIBC progress and eventually develop locally advanced unresectable or metastatic disease, which is associated with high morbidity and mortality rates. The standard of care (SOC) treatment for MIBC is cisplatin-based neoadjuvant chemotherapy (NAC) alone or with immunotherapy (durvalumab, NIAGARA), followed by radical cystectomy (RC). NAC has been shown to both downstage tumors and improve recurrence-free survival (RFS) and disease-specific survival (DSS) [1,2]. Unfortunately, many patients are cisplatin-ineligible (CI) due to medical comorbidities, such as underlying kidney disease, peripheral neuropathy, hearing loss and/or cardiac disease, or poor performance status. CI-MIBC patients typically undergo radical cystectomy, enroll in a clinical study, or receive a combination of gemcitabine and carboplatin (GCa) before definitive surgery [3]. However, the response rates for GCa were lower than those to SOC cisplatin-based NAC. International guidelines provide Grade A recommendations against non-cisplatin-containing regimens in CI patients and recommend proceeding directly with RC [4]. Thus, a significant portion of MIBC patients do not receive effective chemotherapy prior to RC, which increases the risk of recurrence and development of metastatic disease. Patients with CI-MIBC are an understudied group with few viable chemotherapeutic options, and few clinical trials are currently open despite the obvious unmet needs.

Recent advances in immuno-oncology (IO) have led to accelerated approval of enfortumab vedotin (EV) for advanced BCa [5]. Early studies in MIBC have shown potential benefits [6]. However, the cost of EV + IO may be prohibitive for many patients with BCa. The cost of EV alone has been estimated to be ~$450,000/year for a single patient, with metastatic cancer patients surviving a median of approximately 13 months [7]. A study examining the cost-effectiveness of EV in the US and UK using probabilistic sensitivity analysis demonstrated that the probability of EV being cost-effective was 0% [8]. EV also carries significant toxicity risks, with black box warnings for potentially fatal skin diseases, including Stevens–Johnson syndrome, toxic epidermal necrolysis, peripheral neuropathy, and cytopenia.

Compelling data from studies in vitro, in vivo, and clinical trials show that pharmacologic concentrations of vitamin C, or ascorbate, has potential as a therapeutic agent for cancer by selectively exerting oxidative damage to cancer cells [9,10,11,12,13,14,15]. High-dose intravenous administration of ascorbate (IVC) can provide high concentrations sufficient to kill cancer cells [12,16,17]. Previous data from our group indicate that IVC is an effective therapeutic agent and yields superior pharmacokinetics with a >100-fold increase in plasma concentrations [18]. Studies in multiple murine cancer models have demonstrated that IVC is safe, synergistically improves carboplatin- and gemcitabine-based chemotherapies [9,19,20,21], has mechanistic plausibility, and affects several cancer tissue markers. In clinical trials, IVC has been shown to have minimal cumulative toxicity, work synergistically with standard chemotherapies, including gemcitabine and carboplatin, and reduce toxicities associated with SOC chemotherapy [9,19,22]. Based on the results of prospective and retrospective studies, IVC may potentiate chemotherapeutic effects, improve quality of life (QOL), and reduce adverse events (AE) in patients receiving chemotherapy. Multiple studies have shown that BCa is sensitive to high-dose ascorbate in vitro and in vivo [12,23].

We undertook a single-arm, Simon design, “window of opportunity” trial enrolling patients with CI-MIBC. Our primary hypothesis was that adding IVC to a single cycle of GCa NAC regimen would significantly improve pathological downstaging at cystectomy compared to historical data with cystectomy alone, and would be well tolerated. We also evaluated QOL metrics. Exploratory translational analyses were also planned to investigate potential molecular correlates of treatment response, contingent upon the availability of sufficient residual tumor tissue at cystectomy.

## 2. Patients and Methods

### 2.1. Study Design, Treatment, and Endpoints

This protocol was designed as an open-label, single-arm, Simon 2-stage trial. The inclusion criteria were age ≥ 18 years; Eastern Cooperative Oncology Group performance status (ECOG) 0–2, adequate bone marrow, liver function, and kidney function preservation confirmed by estimated or calculated GFR ≥ 30 mL/min, and normal Glucose-6-phosphate dehydrogenase (G6PD) status. Trial-specific exclusion criteria included current consumption of tobacco products (patients were asked to quit for two weeks prior to enrollment and confirmed by blood nicotine levels) and a known history of G6PD deficiency. Based on the Simon 2-stage design, toxicity and efficacy were evaluated after the first stage. Safety was evaluated using the revised NCI Common Terminology Criteria for Adverse Events (CTCAE) version 5.0. If >20% of subjects had grade 3 or 4 toxicity, probably or directly related to IVC, further enrollment would be put on hold for toxicity analysis.

Consenting participants received a single cycle of gemcitabine (1000 mg/m^2^, days 1 and 8)/carboplatin (AUC 5, day 1) chemotherapy, and IVC treatment was started at a dose of 25 g/infusion and titrated to achieve 350–400 mg/dL blood concentrations (up to 125 g/infusion was used). IVC was then given 2 × weekly for 3 weeks. Cystectomy was performed at 4–6 weeks from treatment initiation, after completion of IVC + GCa. The primary endpoint of the study was the pathological stage assessed at cystectomy. Secondary endpoints included QOL metrics using the Functional Assessment of Cancer Therapy-Bladder (FACT-Bl), disease-free survival (DFS), and disease-specific survival (DSS).

### 2.2. Clinical Trial Statistical Analysis

Simon’s two-stage minimax design was used in this study. We defined a response as downstaging to noninvasive disease (<ypT2N0Mx). With the assumption of a successful first stage with one or more responses in 12 patients based on an anticipated downstaging of 8% without NAC [24], we planned to accrue 21 subjects. Descriptive statistics were calculated and provided for all collected data (i.e., demographic and study-specific measures). These included the mean, median, standard deviation, and histograms for all quantitative values, and frequency tables for categorical values. Informed consent and adverse event rates were calculated, and 95% confidence intervals were generated to provide valuable information for future studies.

QOL, as measured by FACT-BI, was analyzed in an exploratory, descriptive manner given the early-phase nature and limited sample size of the trial. Summary statistics (mean, standard deviation, and median) were computed for each subscale and the total score at baseline, end of treatment (EOT), and 6 weeks post-cystectomy. Within-patient changes from baseline to EOT and from baseline to post-surgery were quantified using paired mean differences and two-sided 95% confidence intervals to assess estimation precision. Exploratory paired *t*-tests with Bonferroni correction were conducted. RFS was evaluated at 1 year, and DSS will be evaluated at 3 and 5 years’ follow-up using the Kaplan–Meier method. Survival curves and median survival time are reported.

### 2.3. Trial Oversight

This study was performed in accordance with the International Ethical Guidelines for Biomedical Research Involving Human Subjects, Good Clinical Practice guidelines, and the Declaration of Helsinki. The Institutional Review Board approved the study protocol, and informed consent documents at the University of Kansas Medical Center (Protocol #: IIT-2019-IVC_CarboGem). All participants provided written informed consent. The trial is listed on the NIH Clinical Trials website https://clinicaltrials.gov/ (accessed on 10 September 2026). Identifier: NCT04046094.

### 2.4. Quality of Life

The participants consented to the QOL questionnaire, FACT-Bl. Questionnaires were completed in the clinic at baseline, at the end of NAC (EOT), and 6 weeks post-cystectomy. The FACT-BL questionnaire incorporates 39 items with five-point Likert scale answers and five subscales: physical well-being, social well-being, emotional well-being, functional well-being, and additional concerns [25]. Scoring and management of missing items were dealt with following the FACIT administration and scoring guidelines [26].

## 3. Results

### 3.1. Patient Enrollment

Sixteen patients consented to participate and were screened. Two patients failed screening and two had rapid disease progression with ECOG status > 3, prior to treatment, which precluded participation. Twelve were ultimately enrolled and completed the study protocol (Table 1). Given the response rate in Stage I, it was determined that further recruitment was unnecessary, as the study goals would be met even if no further patients demonstrated a response in Stage 2. The mean age at enrollment was 76.3 ± 9.4 years, with a 3:1 male-to-female ratio. The demographic data in this study mirror the SEER population data on bladder cancer distribution [27]. Eleven patients had transitional cell carcinoma (TCC) and one had TCC with plasmacytoid variant histology. The cohort characteristics are shown in Table 1.

### 3.2. Efficacy

Clinically significant activity and tumor downstaging (<ypT2N0Mx) were achieved in four out of 12 participants (33%, two-sided Clopper–Pearson confidence interval 9.92%, 65.11%) with complete response (CR, ypT0N0Mx) noted in three participants (25%), including one with locally advanced plasmacytoid variant histology, which is known to be highly aggressive and resistant to treatment. This patient remained disease-free through 18 months of follow-up. Comparing the pathological downstaging rates from a single cycle of GCa + IVC to expected rates of 6–8% from TUR alone [1,2,24], our data showed significant improvement (two-sided exact binomial test *p* = 0.008).

**Figure 1:**
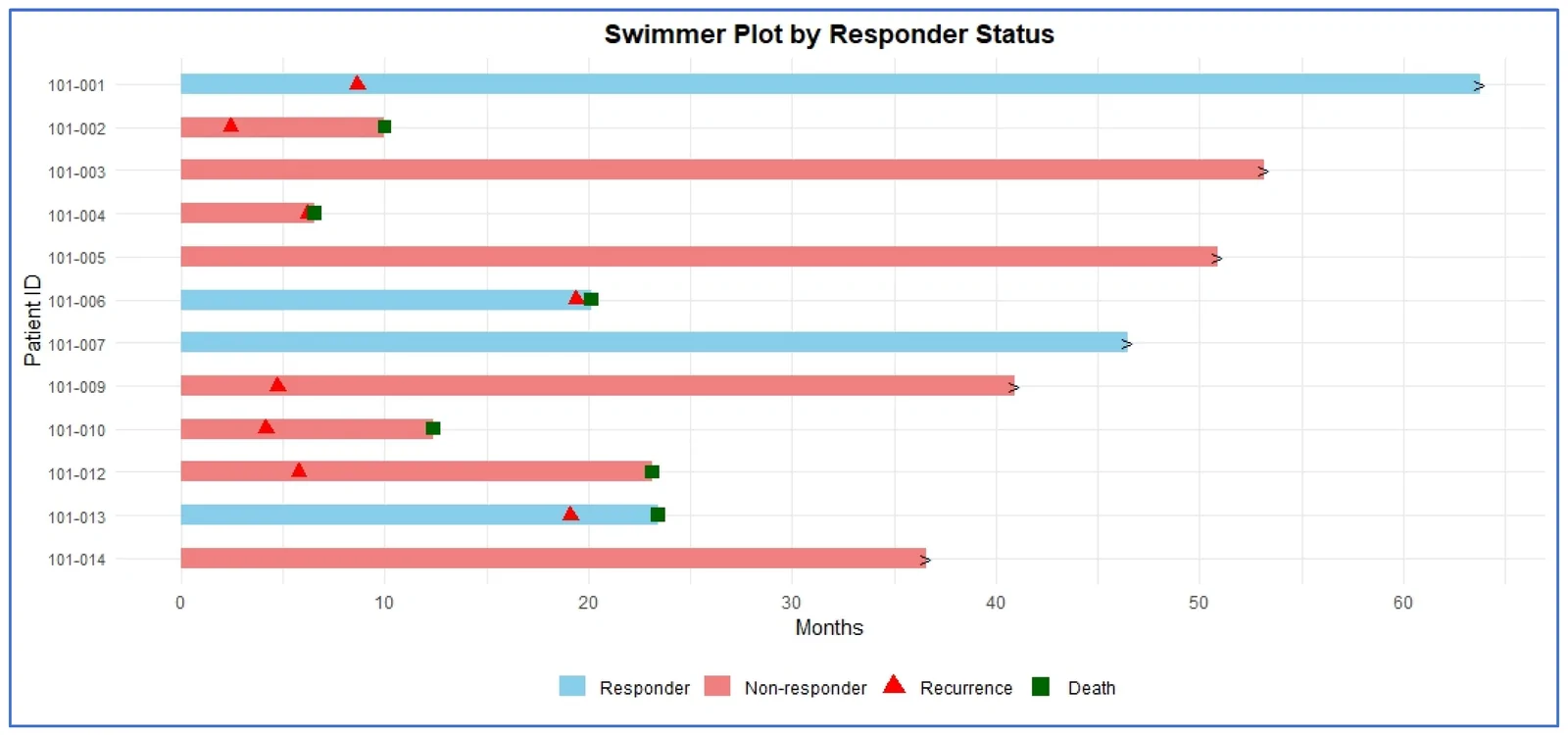
Swimmer plot of trial participants.

For the 12 participants, the median recurrence-free survival (RFS) was 13.9 months (95% CI: 5.8 months—not estimable), and the median overall survival (OS) was 23.4 months (95% CI: 20.1 months—not estimable), as estimated by Kaplan–Meier analysis. The 95% CI upper bounds for both RFS and OS were not estimable because of limited events and censoring beyond this time point. The RFS and OS between responders and non-responders were visually illustrated using a swimmer plot (Figure 1).

Of the four responders, three experienced tumor recurrence. One patient then received systemic immunotherapy and achieved a CR; one died of disease progression, and the other died of unrelated causes. Of the eight non-responders, five recurrences with three disease-related deaths were observed.

Kaplan–Meier analyses were performed separately for responders and non-responders to estimate RFS and OS. For the four responders, the median RFS was 19.3 months (95% CI: 8.6 months—not estimable), and the median OS was 23.4 months (95% CI: 20.1 months—not estimable). For the eight non-responders, median RFS was 6.0 months (95% CI: 4.7 months—not estimable), and median OS was 23.1 months (95% CI: 12.4 months—not estimable). The upper limits of the 95% confidence intervals for median RFS and OS were not estimable because of limited events or censoring beyond the observed median times. These survival estimates should be interpreted with caution because of the small sample size, which limits the precision and generalizability of the results.

The mean time from the start of the study treatment to radical cystectomy was 42.6 ± 13 days. No treatment-related delay was observed in the time to cystectomy (TTC). The IVC + GCa regimen was well tolerated. All 12 patients completed the therapy with no treatment-related AEs or SAEs.

### 3.3. Quality of Life

At baseline, 11 participants completed the FACT-Bl and were evaluated. At EOT and prior to surgery, seven participants completed the FACT-Bl and were compared to baseline. At the post-surgery 6-week follow-up, five subjects completed the FACT-Bl and were compared to baseline (Table 2). Due to attrition and the small cohort size, these outcomes were evaluated descriptively. The mean paired differences and 95% confidence intervals from baseline to each follow-up are reported in Table 2. Among evaluable respondents, overall scores and all five subscales remained numerically stable from baseline to EOT to the 6-week post-surgery assessment. To assess potential attrition bias, baseline characteristics and clinical outcomes were compared between completers and non-completers (Supplementary Tables S1 and S2). At EOT, completers (*n* = 7) and non-completers (*n* = 4) showed comparable, baseline FACT-Bl total scores, recurrence rates, and mortality, with a nominally higher baseline social well-being score among non-completers (26.9 vs. 24.1, *p* = 0.037; non-significant after multiplicity adjustment). Similarly, at post-cystectomy, completers (*n* = 5) and non-completers (*n* = 6) exhibited similar baseline total QOL and subscales, response rates, recurrence, and overall mortality. Because data were limited to surviving and evaluable respondents, these descriptive findings are encouraging but should not be interpreted as cohort-wide absence of functional decline. As decreased QOL at EOT was observed with chemo/radiation therapies in MIBC patients [28,29], the effects of Gem/Carbo + IVC NAC on the QOL of patients with MIBC should be further investigated in larger clinical trials.

## 4. Discussion

Patients with CI-MIBC have limited options for NAC, which contributes to poor prognosis and overall survival. Although new agents, including immune checkpoint inhibitors and/or antibody drug conjugates, are currently being investigated in clinical trials, it is yet to be demonstrated that these regimens have clear benefits for this patient population. In addition, additional treatment-related toxicities, cost-effectiveness, and possible delays in the time to definitive surgery are challenges [30].

This Phase Ib/IIa translational study demonstrated that high-dose IVC combined with a single cycle of GCa demonstrates efficacy as an NAC option for CI-MIBC patients. While this was designed as a Simon two-stage study, we exceeded our efficacy endpoints in Stage 1, with pathological downstaging (<ypT2N0Mx) present in 4/12 (33%) patients. More impressively, three of these four patients (75%) had a CR (ypT0NoMx), including one patient with locally advanced plasmacytoid variant histology, which is particularly notable given the extremely aggressive nature of this variant and typical resistance to platinum-based therapy [31,32]. The treatment was well tolerated with no attributable AE/SAEs. At a mean follow-up of 32.3 ± 18.9 months, an RFS of 13.9 months was noted for the study cohort. There was a clinically significant difference between the responders (19.3 months) and non-responders (6 months); however, the sample size precludes definitive estimates.

Although the study met its prespecified Stage 1 efficacy threshold, the planned second stage was not initiated because the COVID-19 pandemic substantially disrupted accrual, prolonged study duration, and increased costs, and sufficient funding was no longer available to complete Stage 2. Consequently, the small sample size limits the precision of the efficacy estimates and underscores the need for validation in a larger cohort. The encouraging safety and pathological response signals observed in this study provided the rationale for an expanded clinical study (NCT06493370), designed to further evaluate this therapeutic approach.

Parenterally administered ascorbate, or IVC, is known to be pharmacologically active against cancer [9,10,11,12,13,14,15,16,17,18,19]. Blood levels from orally administered ascorbate are tightly controlled by gastrointestinal tract absorption, whereas intravenous administration bypasses this tight control, resulting in markedly higher systemic concentrations [10,11,33]. These higher concentrations produce well-documented effects that are not observed following oral administration. Specifically, the formation of ascorbyl radicals and hydrogen peroxide makes it a pro-oxidative therapy [10,11,12,13].

IVC therapy may be beneficial in patients who are not eligible for the current SOC therapy. Profound safety and low toxicity have been documented with high-dose IVC. A recent pharmacokinetic study [18] showed that IVC was safe at high doses, similar to those used in this study, and did not cause AE/SAEs. There was no evidence of renal or other organ injuries. Previous clinical trials with doses of 75 g/kg and higher IVC have also demonstrated safety in patients with cancer. This study confirmed these findings, as no attributable AE/SAEs were identified.

Previous translational research from our group and others indicates that IVC is synergistic with multiple therapeutic modalities, including chemotherapy, radiation therapy, and immunotherapy [9,13,14,19,20,21,22,34,35], through mechanisms involving increased oxidative damage to cancer cells, modulation of cancer immunity, and alternations in tumor microenvironment. Preclinical and clinical evidence further supports that IVC enhances gemcitabine and carboplatin treatment efficacy [9,19]. This may provide an option for patients with CI-MIBC who are not eligible for standard NAC. The multiple mechanisms of action underlying IVC’s anti-tumor effects also provide a rationale for investigating IVC as an adjunct to other anticancer therapies.

Retrospective studies have shown that time to radical cystectomy > 10 weeks was associated with reduced overall survival and cancer-specific survival in patients with MIBC [30]. Treatment with NAC for MIBC usually involves 3–4 cycles of cisplatin-based combination chemotherapy. An accelerated regimen using dose-dense MVAC allowed for the delivery of chemotherapy over a shorter duration of 6–8 weeks compared to 12–16 weeks with cisplatin and gemcitabine. This allowed patients to undergo earlier definitive surgery without compromising efficacy or safety [36]. In the current trial, we studied a single cycle of carboplatin and gemcitabine chemotherapy with IVC without delaying the time to definitive surgery in CI patients. This study may serve as a blueprint for the evaluation of novel chemotherapy, immunotherapy agents, or antibody–drug conjugates in the neoadjuvant setting.

The largest randomized prospective QOL analysis in patients with MIBC using the FACT-Bl has been recently published [37]. In the BC2001 trial, FACT-Bl was administered at baseline, end of treatment (EOT), 6 months, 1 year, and yearly thereafter up to 5 years. Our trial administered the FACT-Bl questionnaire at baseline, EOT, and six weeks after cystectomy. The BC2001 QOL showed a reduction in QOL at EOT, consistent with treatment-related toxicity that rebounded at 1 year. In comparison, we reported no reduction in QOL at EOT compared to baseline and had no significant treatment-related toxicities (Table 2). The combination of IVC and NAC was well tolerated, with participants completing therapy without changes in QOL. However, a comparison between trials is challenging. The current reported trial is a small proof-of-concept trial, while the BC2001 trial is large. The BC2001 trial was also bladder-conserving, combining radiation in all arms with or without chemotherapy. They did not find intergroup QOL differences and felt that treatment-related toxicity was likely radiation-related. Our trial did not use radiation therapy. In addition, all patients in the study underwent ileal conduit urinary diversion; therefore, differences in QOL attributable to diversion choice were not a factor within this cohort. QOL assessments in this study were limited to 6 weeks after surgery, whereas many differences in HRQOL associated with urinary diversion type, including those related to orthotopic neobladder in older patients, become more apparent over longer-term follow-up. We propose to overcome these concerns with a larger trial and incorporate FACT-Bl.

We attempted to identify early markers that may indicate a successful response to therapy. However, due to the small sample size of the trial and the high number of CRs, tissue-based biomarker analysis was not feasible because of minimal or no residual tumor tissue to make comparisons between responders and non-responders. Future studies with larger sample sizes should investigate the differences between pretreatment and RC specimens using matched sample biomarkers when available.

The primary limitation of this study was its small sample size. However, it was designed as a proof-of-concept study and intended to guide a larger randomized trial if indicated. While the study was designed as a Simon two-stage, we met and exceeded our initial point of continuation in the first stage, precluding stage 2, given the high response rate indicative of significant clinical activity. We also acknowledge the need for more diversity in our participant population, although demographically, our patient population was consistent with that found in BCa. Also, patient attrition in QOL evaluation constrained the exploratory analysis to 7/11 participants at end of treatment (EOT) and 5/11 post-cystectomy. Reassuringly, descriptive comparisons between completers and non-completers revealed no systematic differences across baseline functional status, pathological response, or survival endpoints, suggesting that missingness was not primarily driven by selective attrition, treatment failure, or inferior clinical outcomes. Prospective assessment of physical function and/or frailty could be incorporated alongside tissue-based biomarkers as potential stratification variables in future studies. A larger trial is ongoing to confirm our findings in a more diverse population (NCT06493370). It will also provide additional material to begin defining the patient population that may benefit the most from this novel treatment.

## 5. Conclusions

Single-cycle Gem-Carbo + HD IVC was well tolerated and showed clinically significant activity in a difficult-to-treat population of CI-MIBC, offering an alternative for patients who otherwise are cisplatin-ineligible and may not realize the benefit of NAC. The response rates observed in this Phase Ib/IIa trial, which is comparable to historical outcomes for SOC therapy, supports the development of a larger trial to confirm these findings, identify biomarkers of response, refine study designs, and identify patients most likely to benefit from therapy.

## Figures and Tables

**Table 1:** Demographics of trial participants and pre- and post-treatment staging data.

IV Vitamin C with Chemotherapy for Cisplatin-Ineligible Bladder Cancer Patients: A Forgotten Group						
	Demographic				Staging (TNM)	
Patient	Race	Ethnicity	Age	Gender	Pre-	Post-
001	White	Non-Hispanic	67	Male	cT2NM0	ypT0N0Mx
002	White	Non-Hispanic	67	Male	cT3N0M0	ypT4N0Mx
003	White	Non-Hispanic	67	Male	cT3N0M0	ypT4N0Mx
004	White	Non-Hispanic	61	Male	cT3N0M0	ypT3N0Mx
005	White	Hispanic or Latino	87	Female	cT2N0M0	ypT2N0Mx
006	Unknown	Unknown	88	Male	cT3N0M0	ypT0N0Mx
007	White	Non-Hispanic	74	Male	cT3N0M0	ypTisN0Mx
009	White	Non-Hispanic	74	Male	cT3NM0	ypT3N0Mx
010	Asian	Non-Hispanic	87	Female	cT4N0M0	ypT3N0Mx
012	White	Non-Hispanic	77	Male	cT3N0M0	ypT3N0Mx
013	White	Non-Hispanic	83	Male	cT4N0M0	ypT0N0Mx
014	White	Non-Hispanic	84	Female	cT3NMx	ypT3N0Mx

**Table 2:** Quality of Life Assessment FACT-Bl.

Baseline vs. EOT (*n* = 7)				
Variable	Baseline (Mean)	EOT (Mean)	Mean Change (95% CI)	*p*-Value
Physical Well-Being	23.6	21.7	−1.98 (−5.98, 2.03)	0.999
Social Well-Being	24.1	21.9	−2.28 (−5.36, 0.80)	0.718
Emotional Well-Being	13.3	15.2	1.91 (−0.09, 3.92)	0.351
Functional Well-Being	16.4	16.3	−0.10 (−6.26, 6.07)	0.999
Additional Concerns	31.0	30.4	−0.57 (−4.63, 3.48)	0.999
TOTAL SCORE	108.4	105.4	−3.01 (−17.15, 11.12)	0.999
**Baseline vs. Post-Surgery (*n* = 5)**				
**Variable**	**Baseline (Mean)**	**Post-Surgery (Mean)**	**Mean Change (95% CI)**	***p*** **-Value**
Physical Well-Being	22.8	23.6	0.80 (−9.49, 11.09)	0.999
Social Well-Being	25.4	21.1	−4.33 (−9.11, 0.46)	0.396
Emotional Well-Being	13.5	17.8	4.28 (−2.41, 10.97)	0.901
Functional Well-Being	17.7	12.6	−5.10 (−14.98, 4.78)	0.999
Additional Concerns	30.2	30.4	0.25 (−8.39, 8.90)	0.999
TOTAL SCORE	109.6	105.5	−4.09 (−37.69, 29.50)	0.999

## Data Availability

The original contributions presented in this study are included in the article. Further inquiries can be directed to the corresponding author.

## Supplementary Materials

The following supporting information can be downloaded at https://www.mdpi.com/article/10.3390/antiox15101318/s1, Table S1: Baseline & clinical comparison of EOT completers vs non-completers; Table S2: Baseline & clinical comparison of post-surgery completers vs. non-completers.
