# Supplementary material for: IV Vitamin C with Chemotherapy for Cisplatin-Ineligible Bladder Cancer Patients: A Forgotten Group
Source: Antioxidants (Basel). 2026 Oct 13;15(10):1318. doi: 10.3390/antiox15101318 (PMC13701584; doi:10.3390/antiox15101318)
Supplement: Supplementary file 1 [file antioxidants-15-01318-s001.zip › antioxidants-4527748-supplementary.pdf]

Supplementary Table S1: Baseline & Clinical Comparison of EOT Completers vs Non-Completers

**Characteristic / Clinical Outcome**	**Completers (n=7)**	**Non-Completers (n=4)**	**p-value**
Age (mean SD)	72.4 (8.4)	80.5 (9.3)	0.174
Baseline TOTAL SCORE (mean SD)	108.4 (23.6)	130.8 (10.1)	0.110
Baseline Physical Well-Being (mean SD)	23.6 (6.2)	26.8 (1.9)	0.365
Baseline Social Well-Being (mean SD)	24.1 (2.0)	26.9 (1.3)	0.037
Baseline Emotional Well-Being (mean SD)	13.3 (7.1)	18.2 (2.2)	0.221
Baseline Functional Well-Being (mean SD)	16.4 (8.4)	23.5 (3.0)	0.140
Baseline Additional Concerns (mean SD)	31.0 (4.8)	35.5 (3.7)	0.144
Responder (n %)	1 (14.3%)	3 (75.0%)	0.088
Recurrence (n %)	4 (57.1%)	2 (50.0%)	>0.999
Death (n %)	2 (28.6%)	1 (25.0%)	>0.999

Supplementary Table S2: Baseline & Clinical Comparison of Post-surgery Completers vs. Non-Completers

**Characteristic / Clinical Outcome**	**Completers (n=5)**	**Non-Completers (n=6)**	**p-value**
Age (mean SD)	75.4 (12.0)	75.3 (7.4)	0.991
Baseline TOTAL SCORE (mean SD)	109.6 (30.8)	122.3 (11.8)	0.370
Baseline Physical Well-Being (mean SD)	22.8 (7.3)	26.4 (1.9)	0.270
Baseline Social Well-Being (mean SD)	25.4 (2.4)	24.9 (2.3)	0.757
Baseline Emotional Well-Being (mean SD)	13.5 (8.1)	16.3 (4.3)	0.477
Baseline Functional Well-Being (mean SD)	17.7 (10.3)	20.0 (5.2)	0.642
Baseline Additional Concerns (mean SD)	30.2 (6.0)	34.6 (2.5)	0.130
Responder (n %)	2 (40.0%)	2 (33.3%)	>0.999
Recurrence (n %)	4 (80.0%)	2 (33.3%)	0.242
Death (n %)	2 (40.0%)	1 (16.7%)	0.545
